# CircCDK17 knockdown inhibits tumor progression and cell glycolysis by downregulaing YWHAZ expression through sponging miR-1294 in cervical cancer

**DOI:** 10.1186/s13048-022-00952-y

**Published:** 2022-02-15

**Authors:** Rui Chen, Fei Liang, Jun Yan, Yu Wang

**Affiliations:** Department of Gynaecology and Obstetrics, Henan Provincial People’s Hospital, Peoples Hospital of Zhengzhou University, School of Clinical Medicine Henan University, No. 7 Weiwu Road Jinshui District, Zhengzhou, 450003 Henan China

**Keywords:** CircCDK17, MiR-1294, YWHAZ, CC

## Abstract

**Background:**

Cervical cancer (CC) is the fourth aggressive tumor affecting women worldwide. Circular RNA (circRNA) is enrolled in CC process. This study aims to unveil the profiles of circ_101119 (circCDK17) in cell proliferation, migration, invasion, apoptosis and glycolysis in CC.

**Methods:**

The expression levels of circCDK17, microRNA-1294 (miR-1294) and tyrosine 3-monooxygenase/tryptophan 5-monooxygenase activation protein zeta (YWHAZ) mRNA were detected by quantitative real time polymerase chain reaction (qRT-PCR). The protein expression levels of YWHAZ, recombinant glucose transporter 1 (GLUT1) and hexokinase 2 (HK2) were determined by western blot. Cell proliferation, migratory and invasive abilities and apoptosis were illustrated by cell counting kit-8 (CCK-8) assay, transwell assay and flow cytometry analysis, respectively. Cell lactate production, glucose uptake and adenosine 5'-triphosphate (ATP) level were severally elucidated by lactate assay kit, glucose assay kit and ATP detection kit.

**Results:**

CircCDK17 expression and the mRNA and protein expression levels of YWHAZ were dramatically upregulated, while miR-1294 expression was obviously downregulated in CC tissues or cells compared with control groups. CircCDK17 silencing suppressed cell proliferation, migration, invasion and glycolysis, and induced cell apoptosis in CC; however, miR-1294 inhibitor restrained these effects. Additionally, circCDK17 was a sponge of miR-1294 and miR-1294 bound to YWHAZ. Furthermore, circCDK17 knockdown inhibited tumor formation in vivo.

**Conclusion:**

CircCDK17 knockdown repressed cell proliferation, migration, invasion and glycolysis, and promoted cell apoptosis via miR-1294/YWHAZ axis in CC. This finding provides a theoretical basis in studying circRNA-mediated therapy in CC.

## Introduction

Cervical cancer (CC) is one of primary reasons of tumor-linked mortalities among women aged 20–39 worldwide [1]. Hundreds of new cases are annually diagnosed in developing or developed countries [2]. Multiple data present that human papillomavirus (HPV) is the main cause of CC [3, 4]. Although radiotherapy can achieve better curative effect in treating advanced CC patients, the recurrence of CC is still high [5]. Therefore, fully understanding the pathogenesis of CC is necessary to seek reliable therapy target.

Circular RNA (circRNA) is a noncoding RNA and is more stable than its matched linear RNA based on its closed loop structure [6]. CircRNA is specifically expressed in cells or tissues, and participates in regulating the biological behavior of human cancers, including CC [7–9]. For example, circ_0000745 was found that it was highly expressed in CC tissues and contributed to cell proliferative and migratory abilities in CC [10]. Hu et al. also indicated that circ_0067934 repression hindered cell proliferation and metastasis in CC [11]. Besides, circ_0005576 was found to be highly expressed in CC tissues and cells, and its silencing suppressed cell proliferation and migratory and invasive abilities in CC [12]. However, at present, the data related to circCDK17 regulating CC process is little.

MicroRNA (miRNA) is a small RNA with about 20 nucleotides in size, and miRNA is widely expressed in cells or tissues. MiRNA mainly acts function via associating with its target gene, which results in mRNA degradation or translation repression [13]. Data implicates that miRNA functions as a tumor promoter or suppressor in cancer progression [14, 15]. Precious researches disclosed that miR-1294 (hsa-miR-1294) inhibited the progression of oral squamous cell carcinoma [16], osteosarcoma [17], hepatocellular carcinoma [18] and so on. Nevertheless, there is few study on CC development regulated by miR-1294.

Tyrosine 3-monooxygenase/tryptophan 5-monooxygenase activation protein zeta (YWHAZ), also named as 14–3-3 zeta, is one of the 14–3–3 gene family [19]. YWHAZ can modulate molecule associating with serine or threonine residue and thereby mediate many biological events, such as cell proliferation, metastasis, glycolysis [20, 21]. In CC, YWHAZ was revealed that it could recuse the influences of long non-coding RNA small nucleolar RNA host gene 14 (SNHG14) on CC process [22], suggesting YWHAZ was enrolled in CC progression.

Herein, the expression levels of circCDK17, miR-1294 and YWHAZ were disclosed in CC tissues and cells. Additionally, recuse experiments were performed to illustrate that circCDK17 regulated cell proliferation, migration, invasion, apoptosis and glycolysis via miR-1294/YWHAZ axis. Furthermore, the influence of circCDK17 silencing on tumor formation of CC was unveiled in vivo.

## Materials and methods

### Specimen acquirement and restore

CC patients from Henan Provincial People’s Hospital provided human CC tissues and human normal cervical tissues with signing the written informed consents. All tissues were instantly kept in refrigerator at -80˚C. The Ethics Committee of Henan Provincial People’s Hospital agreed with this research.

### Cell purchase and culture

Human CC cell lines (C-33A and HeLa) and human cervical epithelial cell line Ect1/E6E7 were obtained from BeNa Culture Collection (Beijing, China). Cells were cultivated in Dulbecco’s modified Eagle’s medium (DMEM; Biosun, Shanghai, China) supplemented with 10% fetal bovine serum (FBS; Biosun) with antibiotics (100 μg/mL penicillin, 100 μg/mL streptomycin) (Thermo Fisher, Waltham, MA, USA) at 37˚C in an incubator with 5% CO_2_.

### Cell transfection

The small interfering RNA against circCDK17 (si-circCDK17), miR-1294 mimic (miR-1294), the overexpression plasmids of circCDK17 (circCDK17) and YWHAZ (YWHAZ), miR-1294 inhibitor (in-miR-1294), the small hairpin RNA targeting circCDK17 (sh-circCDK17) and controls (si-NC, miR-NC, Vector, pcDNA, in-miR-NC and sh-NC) were synthesized by GENEWIZ (Suzhou, China). Cell transfection was carried out with Lipofectamine 3000 (Thermo Fisher) according to manufacturer’s instructions. The synthesized sequences were si-circCDK17 5’-ATGGAAGCAGATTAGGATTTT-3’, miR-1294 mimic 5’-UGUGAGGUUGGCAUUGUUGUCU-3’, miR-1294 inhibitor 5’-AGACAACAAUGCCAACCUCACA-3’, si-NC5’-CCACAAATGTAATGGTCTT-3’, miR-NC 5’-UUUGUACUACACAAAAGUACUG-3’ and in-miR-NC 5’-CAGUACUUUUGUGUAGUACAAA-3’.

### Quantitative real time polymerase chain reaction (qRT-PCR)

CC tissues and cells were lysed using TransZol (TransGen, Beijing, China). And RNA was extracted with RNAsimple kit (Tiangen, Beijing, China). RNA concentration was measured with NanoDrop-1000 apparatus (Thermo Fisher). cDNA was synthesized using High-Capacity RT Kit (Thermo Fisher) or TQiagen RT kit (Hilden, Germany). For quantifying the amount of circCDK17, miR-1294 and YWHAZ, SuperReal PreMix Color (Tiangen) was performed. Data was analyzed with the 2^−∆∆Ct^ method with U6 or β-actin as control. The forward and reverse primers were circCDK17 5’-GCCCAGAAATGGAAGCAG-3’ and 5’-TTATCCTTGCTGCTGTTT-3’; cyclin dependent kinase 17 (CDK17) 5’-GCCTAACAAACTGCTGTTCTTTCT-3’ and 5’-CACAGCCTTCATCCCCAAGA-3’; miR-1294 5’-CTCACGAGAGAGGAAGGCA-3’ and 5’-ACCTCAAGAACAGTATTTCCAGG-3’; YWHAZ 5’-AGCTGGTTCAGAAGGCCAAA-3’ and 5’-AAGATGACCTACGGGCTCCT-3’; U6 5’-TGCGGGTGCTCGCTTCGGCAGC-3’ and 5’-GTGCAGGGTCCGAGGT-3’; β-actin 5’-CACCATTGGCAATGAGCGGTTC-3’ and 5’-AGGTCTTTGCGGATGTCCACGT-3’.

### RNase R treatment assay

C-33A and HeLa cells were collected and RNA was extracted in the same manner as above. Extracted RNA was incubated with RNase R^+^ (Epicentre, Madison, WI, USA) at a dose of 3U/μg RNA at 37˚C for 30 min. After that, RNeasy MinElute Cleaning Kit (Qiagen, Valencia, CA, USA) was performed to purify RNA. The amount of circCDK17 was determined by qRT-PCR with linear CDK17 as a control.

### Cell counting kit-8 (CCK8) assay

The proliferation of C-33A and HeLa cells was illustrated by CCK-8 assay. In short, C-33A and HeLa cells were grown in 96-well plate for 18 h. Following plasmid, miR-1294 mimic or miR-1294 inhibitor was transfected into cells with their controls and cells were continued to culture for 0, 24, 48 and 72 h. Medium was removed and CCK-8 solution (Beyotime, Shanghai, China) was added into well. 2 h later, the absorbance at 450 nm was detected with microplate reader (Thermo Fisher).

### Transwell assay

Cell migration and invasion were revealed using transwell chamber (8.0 µm) without and with Matrigel (Corning, Madison, New York, USA), respectively. In brief, cells were mixed with FBS-free DMEM (Biosun) and then added into upper chamber of 24-well plate. DMEM contained 15% FBS was placed into lower chamber. After 24 h, supernatant was discarded and cells were washed using phosphate buffer solution (PBS) (Thermo Fisher). Then methanol (Beyotime) and crystal violet (Beyotime) were severally employed to immobilize and stain cells. The migratory and invasive abilities were determined by counting cell numbers with microscope (Olympus, Tokyo, Japan) with a 100( ×) magnification.

### Flow cytometry analysis

Cell apoptosis was analyzed by Annexin V-fluorescein isothiocyanate (Annexin V-FITC) detection kit (Yeasen Biotech, Shanghai, China). Briefly, cells were collected after digested using trypsin (Thermo Fisher). Cells were washed twice with PBS (Thermo Fisher) and precipitated by centrifuging at 300 g for 6 min. Then cells were suspended in binding buffer (Yeasen Biotech). Annexin V-FITC (Yeasen Biotech) and propidium iodide (PI) (Yeasen Biotech) were used to incubate cells, respectively. Cell samples were assessed by flow cytometry (BD Biosciences, San Diego, CA, USA).

### Lactate production and glucose uptake analysis

Lactate production and glucose uptake were determined by lactate assay kit (Abcam, Cambridge, UK) and glucose assay kit (Abcam), respectively. In short, cells were collected and PBS (Thermo Fisher) was then employed to wash cells. Then cells were suspended in lactate/glucose assay buffer (Abcam). Cells were centrifuged at 1,3400 g for 2 min and supernatant was collected. Enzyme in samples was removed using Deproteinizing Sample Preparation Kit (Abcam). Results were demonstrated by measuring absorbance at 450 nm or 570 nm using microplate reader (Thermo Fisher).

### Adenosine 5'-triphosphate (ATP) detection assay

ATP production was detected with ATP detection kit (Beyotime). Cells were collected and lysed using lysis buffer (Beyotime). Following supernatant was obtained via centrifuging at 1,2000 g for 6 min. And ATP detection reagent (Beyotime) was added. 3 min later, samples and standard samples were severally placed into test well. Samples were assessed by luminometer (Promega, Madison, WI, USA).

### Western blot analysis

Cells and tumor tissues were lysed using RIPA buffer (Beyotime). Lysates were mixed with loading buffer (Thermo Fisher), which was then boiled in boiling water. Then samples were loaded on 12% bis–tris-acrylamide gel (Thermo Fisher) and then electrotransferred onto polyvinylidene fluoride (Millipore, Bradford, MA, USA). After immersed in 5% nonfat milk diluted with tris buffered saline tween (Millipore), membranes were incubated with anti-recombinant glucose transporter 1 (anti-GLUT1) (1:200; Abcam), anti-hexokinase 2 (anti-HK2) (1:1000; Abcam), anti-YWHAZ (1:1000; Abcam) and anti-β-actin (1:1000; Abcam). Secondary antibody labeled with horseradish peroxidase (1:10,000; Abcam) was subsequently used to incubate membranes. Protein bands were visualized using RapidStep ECL Reagent (Millipore). β-actin acted as a control.

### Cytoplasmic and nuclear RNA analysis of circCDK17

The cell location of circCDK17 was analyzed with PARIS™ Kit (Thermo Fisher). In short, C-33A and HeLa cells were collected and washed using PBS (Thermo Fisher). And cells were suspended in cell fractionation buffer (Thermo Fisher) for 8 min. Then samples were centrifuged at 500 g for 4 min to separate cytoplasmic fraction from nuclear pellet. Following cytoplasm and nuclear pellet were lysed and RNA was extracted according to procedures descripted as above. CircCDK17 expression was assessed using qRT-PCR with β-actin and U6 as references.

### Dual-luciferase reporter assay

The binding sites between miR-1294 and circCDK17 or YWHAZ were predicted by interactome or targetscan online database. The wild-type (WT) plasmids of circCDK17 (circCDK17 WT) and YWHAZ (YWHAZ 3’UTR WT) were built via inserting the sequences of circCDK17 and YWHAZ 3’UTR containing the putative binding sequences of miR-1294 into pmirGLO (Promega). The target sites of miR-1294 in circCDK17 and YWHAZ 3’UTR were mutated and mutant (MUT) vectors of circCDK17 (circCDK17 MUT) and YWHAZ (YWHAZ 3’UTR MUT) were established in the same manner as above. Cell transfection was performed and luciferase activities were detected according to manufacturer’s instructions of Dual-Lucy Assay Kit (Solarbio, Beijing, China). *Renilla* Luciferase activity was employed to as an internal control of firefly luciferase activity.

### RNA pull-down assay

Oligonucleotide probe targeting circCDK17 was synthesized by RiboBio (Guangzhou, China). C-33A and HeLa cells were lysed and incubated with probes with biotin (Probe-biotin) and without biotin (Probe-no biotin), which was then incubated with Streptavidin-coupled Dynabeads (Invitrogen, Carlsbad, CA, USA). After that, captured complexes were incubated with RIP buffer possessing proteinase K (Millipore). The amount of circCDK17 and miR-1294 was detected by qRT-PCR.

### Xenograft mouse model assay

Female BALB/c nude mice (5-week old) were obtained from Charles River (Beijing, China). Mice were randomly divided into 2 groups (sh-NC group and sh-circCDK17 group, N = 5 per group). 5 × 10^6^ HeLa cells stably transfected with sh-circCDK17 or sh-NC were intraperitoneally injected into nude mice. Tumor volume was measured every 7 days. 28 days later, mice were euthanized and tumors were excised. In order to detect the effects of circCDK17 on the expression levels of miR-1294 and YWHAZ, a part of tumors was kept in refrigerator at -80˚C. The Animal Care and Use Committee of Henan Provincial People’s Hospital approved this study. Guide for the Care and Use of Laboratory Animals was strictly followed.

## Data analysis

Data was obtained based on 3 replicates and analyzed with SPSS 21.0 software (IBM, Somers, NY, USA). Results were presented as means ± standard deviations (*SD*). Significant differences were compared with two-tailed Student’s t-tests, Wilcoxon rank-sum test or one-way analysis of variance (ANOVA). *P* value < 0.05 was considered statistically significant.

## Results

### CircCDK17 was highly expressed in CC tissues and cells

**Fig. 1:**
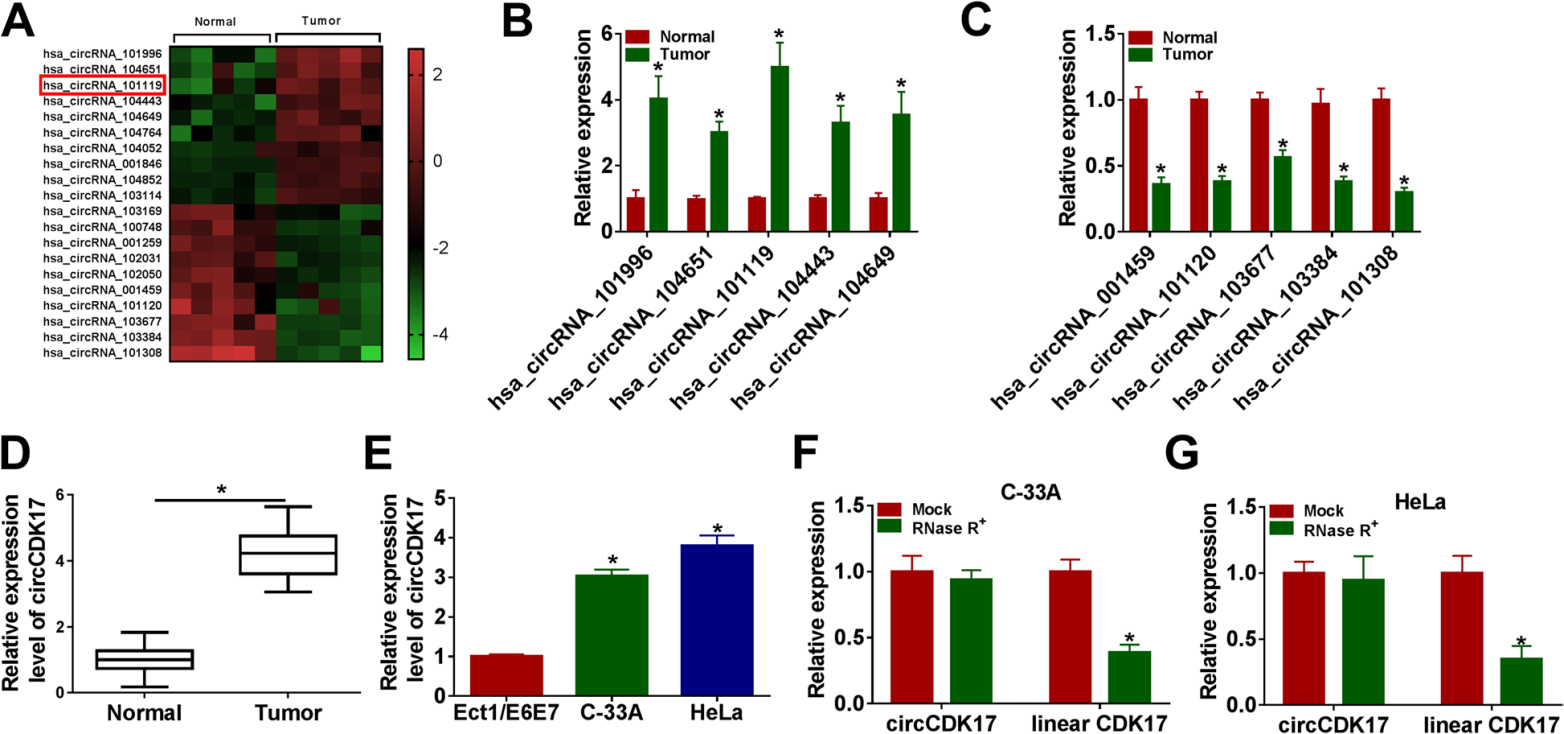
CircCDK17 expression was dramatically upregulated in CC tissues and cells.** A** GSE102686 database showed differently expressed circRNAs in CC tissues and normal cervical tissues. **B** and **C** The top 5 upregulated and 5 downregulated circRNAs were assessed by qRT-PCR in CC tissues and normal cervical tissues. **D** and **E** CircCDK17 expression was detected by qRT-PCR in CC tissues, normal cervical tissues, Ect1/E6E7 cells and C-33A and HeLa cells. **F** and **G** RNase R^+^ treatment assay was performed to investigate circCDK17 was a circular RNA. *P < 0.05

In order to seek important circRNA in regulating CC progression, GSE102686 database was employed. And the top 10 upregulated and 10 downregulated circRNAs were displayed in Fig. 1A. Subsequently, the expression levels of top 5 upregulated and 5 downregulated circRNAs in CC tissues were analyzed by qRT-PCR. Results showed that there was the most obvious change in circ_101119 (named as circCDK17) expression in CC tissues compared with normal cervical tissues (Fig. 1B and C). QRT-PCR further illustrated that circCDK17 expression was significantly upregulated in CC tissues and C-33A and HeLa cells relative to normal cervical tissues and Ect1/E6E7 cells, respectively (Fig. 1D and E). Additionally, results illustrated that circCDK17 expression was not apparently changed after RNase R^+^ treatment, whereas linear CDK17 expression was obviously downregulated in C-33A and HeLa cells (Fig. 1F and G). These results meant that circCDK17 might play a vital role in CC process.

### CircCDK17 knockdown repressed cell proliferation, migration, invasion and glycolysis, and induced cell apoptosis in CC

**Fig. 2:**
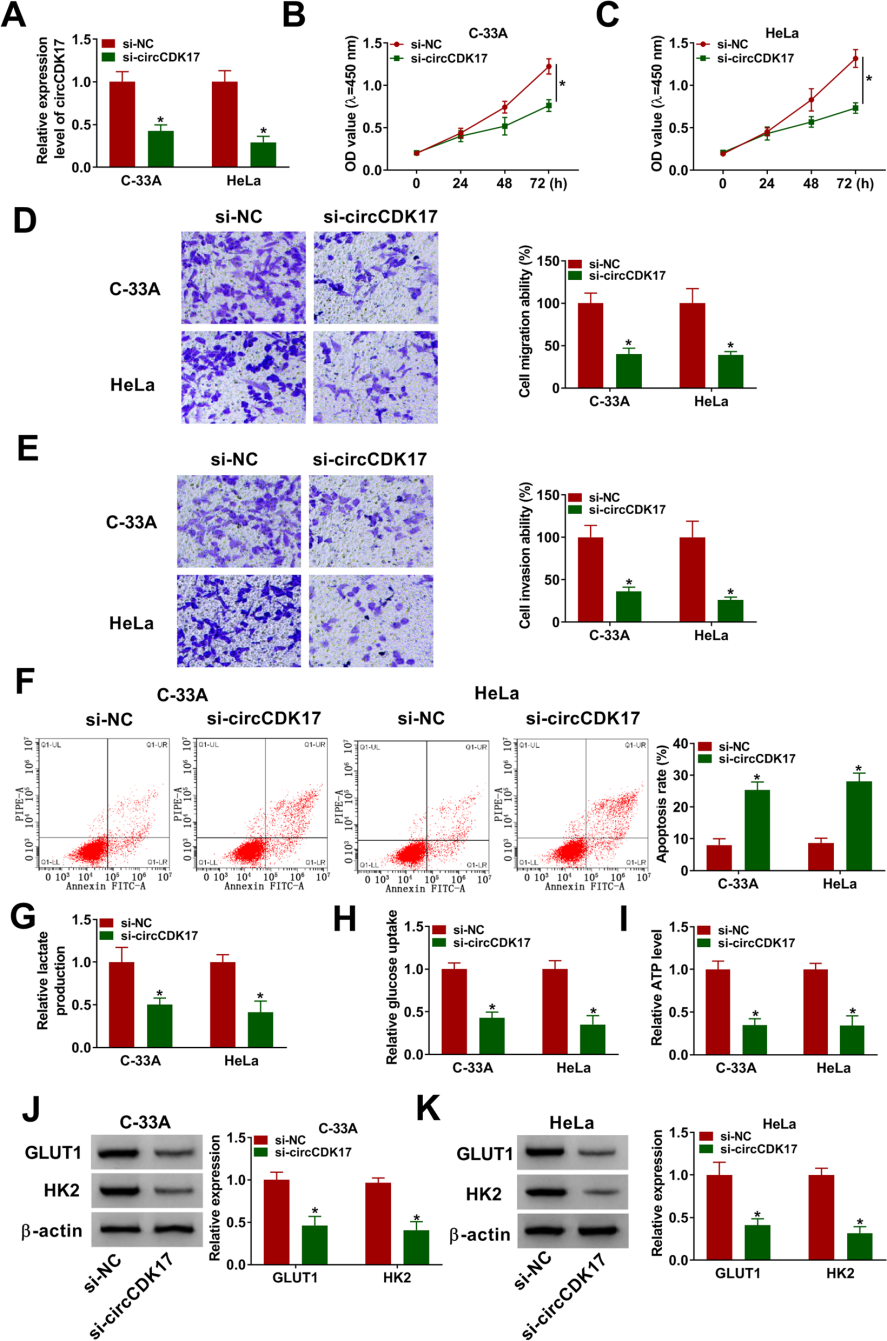
CircCDK17 silencing inhibited tumor development and glycolysis in CC.** A** The transfection efficiency of si-circCDK17 was determined by qRT-PCR in C-33A and HeLa cells. **B** and **C** The effect of circCDK17 knockdown on cell proliferation was illustrated by CCK-8 assay in C-33A and HeLa cells. **D** and **E** The impacts of circCDK17 silencing on the migration and invasion of C-33A and HeLa cells were demonstrated by transwell assay. **F** Flow cytometry analysis was performed to investigate the influence of circCDK17 knockdown on the apoptosis of C-33A and HeLa cells. **G** Lactate assay kit was employed to the influence of circCDK17 silencing on lactate production in C-33A and HeLa cells. **H** Glucose assay kit was used to determine the impact of circCDK17 knockdown on glucose uptake in C-33A and HeLa cells. **I** ATP detection kit was conducted to present the effect of circCDK17 silencing on ATP level in C-33A and HeLa cells. **J** and **K** Western blot was employed to illustrate the effects of circCDK17 downregulation on the protein expression levels of GLUT1 and HK2 in C-33A and HeLa cells. *P < 0.05

The function of circCDK17 in CC process was continued to explore. The interfering plasmid of circCDK17 was firstly built. And data presented that circCDK17 expression was dramatically downregulated after circCDK17 silencing in C-33A and HeLa cells related to control groups (Fig. 2A). Subsequently, results showed that circCDK17 silencing suppressed cell proliferation in C-33A and HeLa cells (Fig. 2B and C). The migratory and invasive abilities of C-33A and HeLa cells were also inhibited in C-33A and HeLa cells transfected with si-circCDK17 compared with control group (Fig. 2D and E). The effect of circCDK17 knockdown on the apoptosis of C-33A and HeLa cells was also explored, and results unveiled that circCDK17 knockdown induced cell apoptosis (Fig. 2F). Additionally, the impact of circCDK17 silencing on glycolysis was disclosed. Results showed that circCDK17 silencing repressed lactate production and glucose uptake and decreased ATP level (Fig. 2G-I). Meanwhile, circCDK17 knockdown dramatically downregulated the protein expression levels of GLUT1 and HK2 in C-33A and HeLa cells (Fig. 2J and K). Thus, the above data presented that circCDK17 knockdown repressed tumor development and glycolysis in CC.

### CircCDK17 acted as a sponge of miR-1294 in C-33A and HeLa cells

**Fig. 3:**
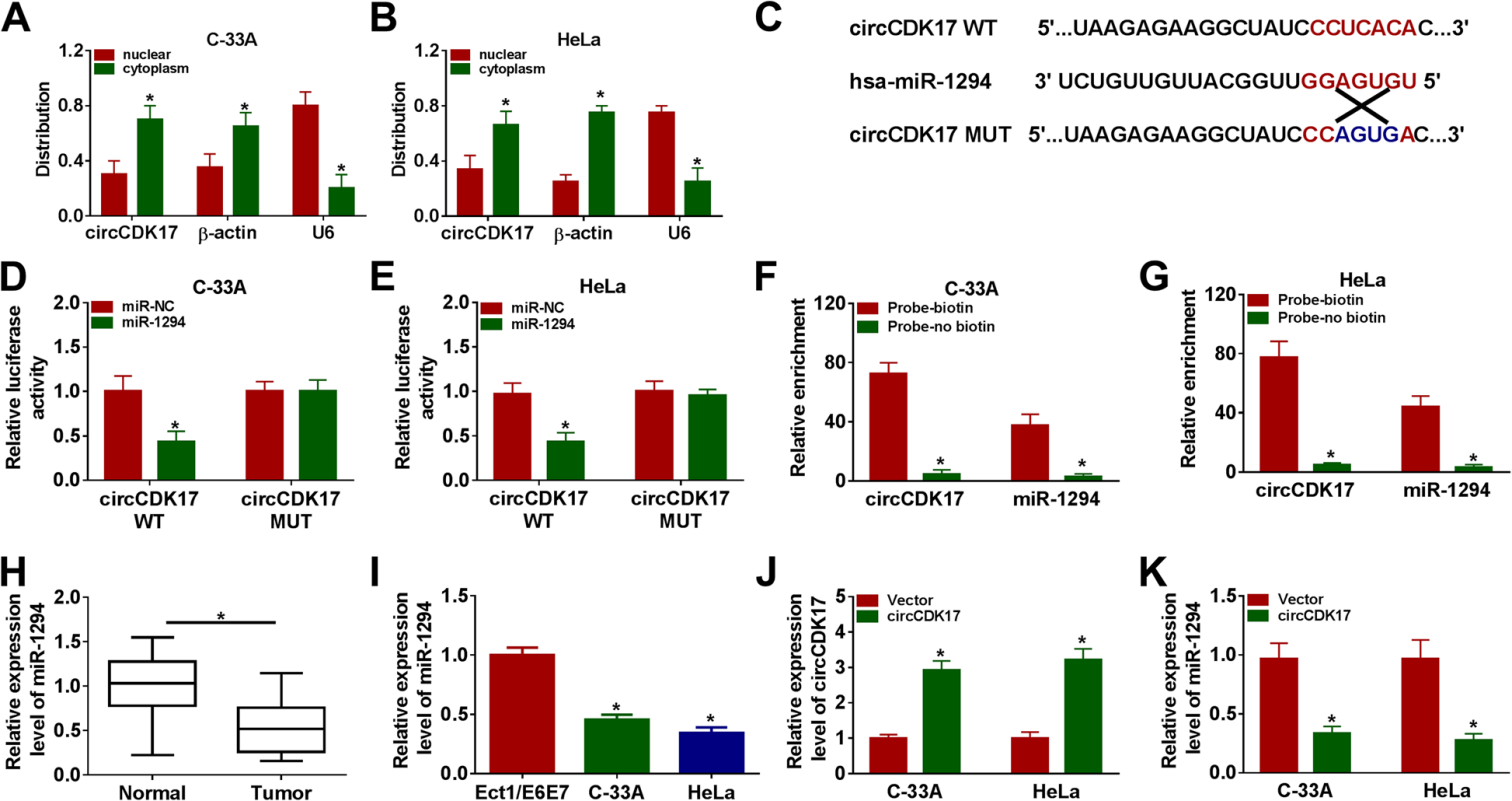
CircCDK17 directly interacted with miR-1294 in CC cells.** A** and **B** Cytoplasmic and nuclear RNA analysis assay demonstrated the cell location of circCDK17, β-actin and U6 in C-33A and HeLa cells. **C** The binding sites between circCDK17 and miR-1294 were predicted by interactome online database. **D** and **E** Luciferase activity was detected by dual-luciferase reporter assay in C-33A and HeLa cells. **F** and **G** RNA pull-down assay was employed to illustrate circCDK17 was directly associated with miR-1294 in C-33A and HeLa cells. **H** and **I** MiR-1294 expression was detected by qRT-PCR in CC tissues, normal cervical tissues and Ect1/E6E7, C-33A and HeLa cells. **J** CircCDK17 expression was determined by qRT-PCR in C-33A and HeLa cells transfected with circCDK17 or vector. **K** QRT-PCR was performed to illustrate the influence of circCDK17 overexpression on miR-1294 expression in C-33A and HeLa cells. *P < 0.05

In order to reveal the underlying mechanism of circCDK17 in regulating tumor development and glycolysis in CC, its cell location was firstly assessed. Results showed circCDK17 expression was dramatical higher in cytoplasm than that in nuclear (Fig. 3A and B). Subsequently, interactome online database demonstrated that circCDK17 contained the binding sites of miR-1294 (Fig. 3C). And results also presented that the luciferase activity of circCDK17 WT and miR-1294 mimic group was obviously repressed, whereas there was no prominent change in circCDK17 MUT and miR-1294 mimic group (Fig. 3D and E). RNA pull-down assay also elucidated that miR-1294 was apparently pulled down by biotin–coupled probe of circCDK17 compared with that by circCDK17 probe without coupling biotin (Fig. 3F and G). Additionally, miR-1294 was found that its expression was apparently decreased in CC tissues and C-33A and HeLa cells as compared to normal cervical tissues and Ect1/E6E7 cells, respectively (Fig. 3H and I). Furthermore, the effect of circCDK17 overexpression on miR-1294 expression was unveiled. Result primarily elucidated that circCDK17 was successfully transfected into C-33A and HeLa cells based on its expression was strikingly upregulated (Fig. 3J). And qRT-PCR result demonstrated that miR-1294 expression was dramatically decreased by enforced circCDK17 expression (Fig. 3K). These data illustrated that circCDK17 was directly associated with miR-1294 in CC cells.

### CircCDK17 silencing repressed tumor development and glycolysis by sponging miR-1294 in CC

**Fig. 4:**
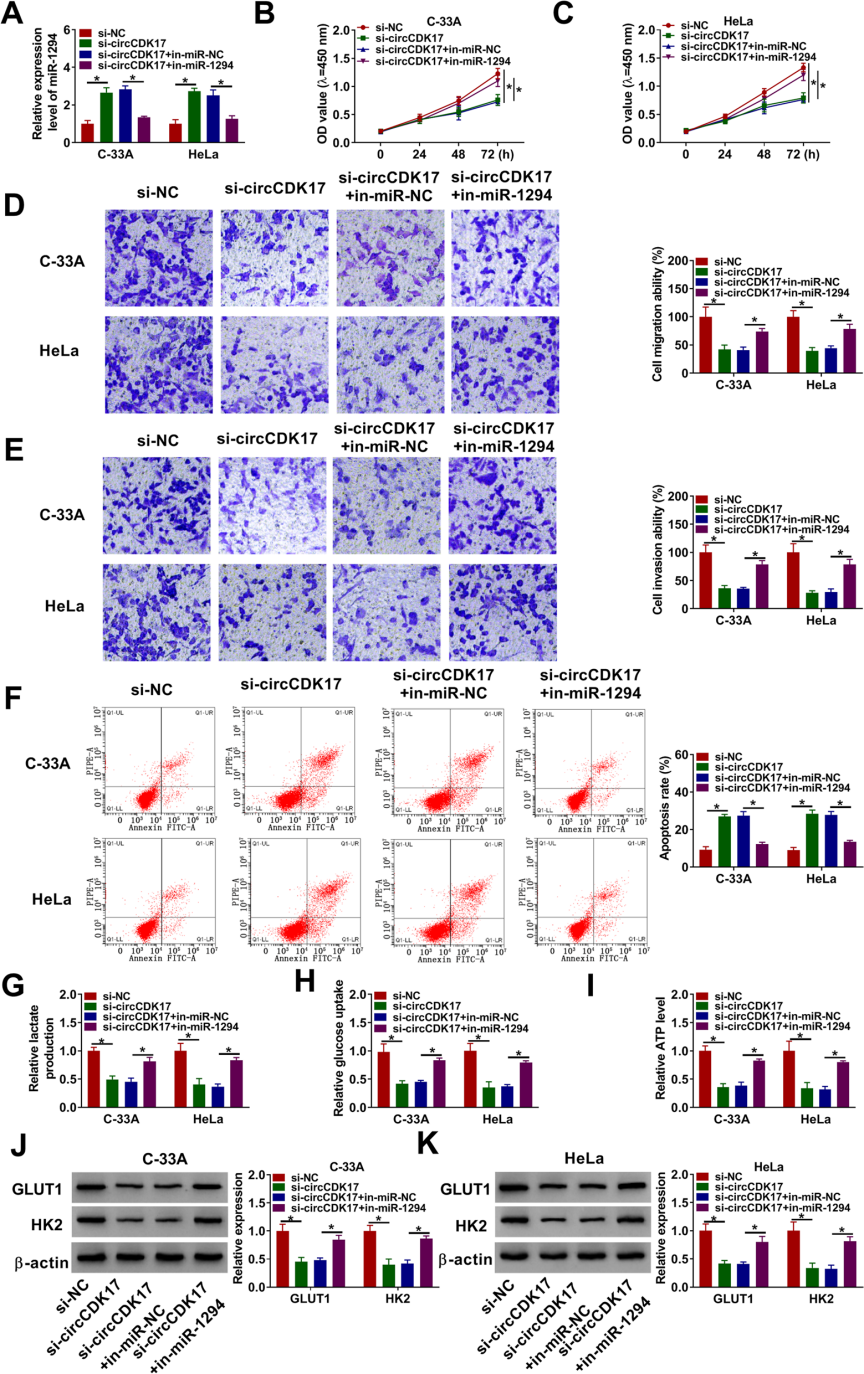
CircCDK17 regulated biological behavior of CC by binding to miR-1294.** A** The effects between circCDK17 repression and miR-1294 inhibitor on miR-1294 expression were investigated by qRT-PCR in C-33A and HeLa cells. **B** and **C** The impacts between circCDK17 knockdown and miR-1294 inhibitor on the proliferation of C-33A and HeLa cells were disclosed by CCK-8 assay. **D** and **E** Transwell assay was performed to illustrate the influences between circCDK17 repression and miR-1294 inhibitor on the migration and invasion of C-33A and HeLa cells. **F** Flow cytometry analysis was employed to elucidate the impacts between circCDK17 knockdown and miR-1294 inhibitor on cell apoptosis in C-33A and HeLa cells. **G** Lactate assay kit was carried out to demonstrate the effects between circCDK17 silencing and miR-1294 inhibitor on lactate production in C-33A and HeLa cells. **H** Glucose assay kit was used to determine the impacts between circCDK17 knockdown and miR-1294 inhibitor on glucose uptake in C-33A and HeLa cells. **I** ATP detection kit was conducted to present the effects between circCDK17 silencing and miR-1294 inhibitor on ATP level in C-33A and HeLa cells. **J** and **K** Western blot was conducted to illustrate the effects between circCDK17 downregulation and miR-1294 repression on the protein expression levels of GLUT1 and HK2 in C-33A and HeLa cells. *P < 0.05

Whether circCDK17 regulated biological behavior of CC via binding to miR-1294 was further studied. Results initially showed that circCDK17 knockdown dramatically upregulated miR-1294 expression, whereas miR-1294 inhibitor attenuated this effect (Fig. 4A). Subsequently, results elucidated that miR-1294 inhibitor restored the inhibition effect of circCDK17 silencing on cell proliferation (Fig. 4B and C). The repression effects of circCDK17 knockdown on the migration and invasion of C-33A and HeLa cells were also abolished by miR-1294 inhibition (Fig. 4D and E). Additionally, data presented that circCDK17 silencing induced the apoptosis of C-33A and HeLa cells, which was relieved after miR-1294 inhibitor transfection (Fig. 4F). The influences between circCDK17 knockdown and miR-1294 inhibitor on glycolysis were further revealed. Results displayed that miR-1294 inhibitor also hindered the inhibitive effects of circCDK17 silencing on lactate production, glucose uptake and ATP level in C-33A and HeLa cells (Fig. 4G-I). And the repressive impacts of circCDK17 silencing on the protein expression levels of GLUT1 and HK2 were restrained after miR-1294 inhibitor transfection (Fig. 4J and K). These evidences elaborated that circCDK17 regulated tumor development and glycolysis via associating with miR-1294 in CC.

### MiR-1294 was associated with YWHAZ in CC cells

**Fig. 5:**
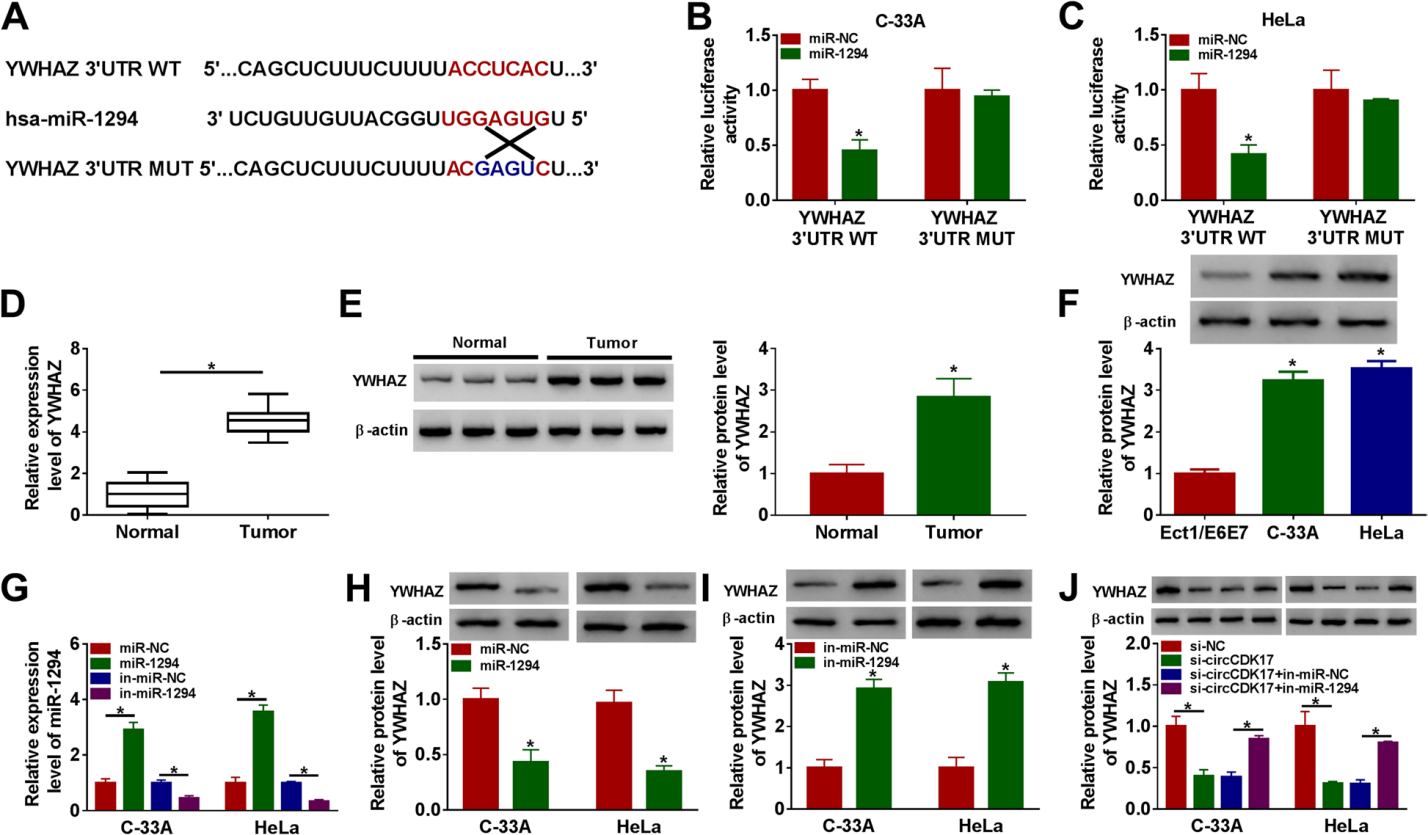
MiR-1294 bound to YWHAZ in CC cells.** A** The binding sites between miR-1294 and YWHAZ were predicted by targetscan online database. **B** and **C** Luciferase activities were detected by dual-luciferase reporter assay in C-33A and HeLa cells. **D**-**F** QRT-PCR and western blot analysis were employed to detect the mRNA and protein expression levels of YWHAZ, respectively, in CC tissues, normal cervical tissues or Ect1/E6E7, C-33A and HeLa cells. **G** The transfection efficiency of miR-1294 mimic and inhibitor was determined by qRT-PCR in C-33A and HeLa cells. **H** and **I** Western blot analysis was performed to present the effects of miR-1294 mimic and inhibitor on YWHAZ protein expression in C-33A and HeLa cells. (J) The impacts between circCDK17 knockdown and miR-1294 inhibitor on YWHAZ protein expression were illustrated by western blot in C-33A and HeLa cells. *P < 0.05

The binding gene of miR-1294 was further explored. Results showed that miR-1294 contained the binding sequence of YWHAZ 3’UTR (Fig. 5A). And dual-luciferase reporter assay also presented that luciferase activity was obviously repressed in YWHAZ 3’UTR WT + miR-1294 mimic group in C-33A and HeLa cells, but that was not apparently changed after YWHAZ 3’UTR MUT + miR-1294 mimic co-transfection (Fig. 5B and C). Subsequently, western blot analysis displayed that the mRNA and protein levels of YWHAZ were obviously upregulated in CC tissues or C-33A and HeLa cells as compared to normal cervical tissues or Ect1/E6E7 cells, respectively (Fig. 5D-F). Additionally, in order to demonstrate the effects of miR-1294 mimic and inhibitor on YWHAZ expression, their transfection efficiency was firstly identified. Data showed miR-1294 expression was apparently upregulated by miR-1294 mimic and was obviously downregulated by miR-1294 inhibitor (Fig. 5G). YWHAZ was then found that its protein expression was strikingly downregulated by miR-1294 mimic and was dramatically upregulated after miR-1294 inhibitor transfection (Fig. 5H and I). Furthermore, western blot results illustrated circCDK17 knockdown significantly decreased YWHAZ protein expression, whereas this effect was attenuated after miR-1294 inhibitor transfection (Fig. 5J). Therefore, our findings demonstrated that miR-1294 interacted with YWHAZ in CC cells.

### MiR-1294 inhibited cell proliferation, migration, invasion and glycolysis, and promoted cell apoptosis by binding to YWHAZ in CC

**Fig. 6:**
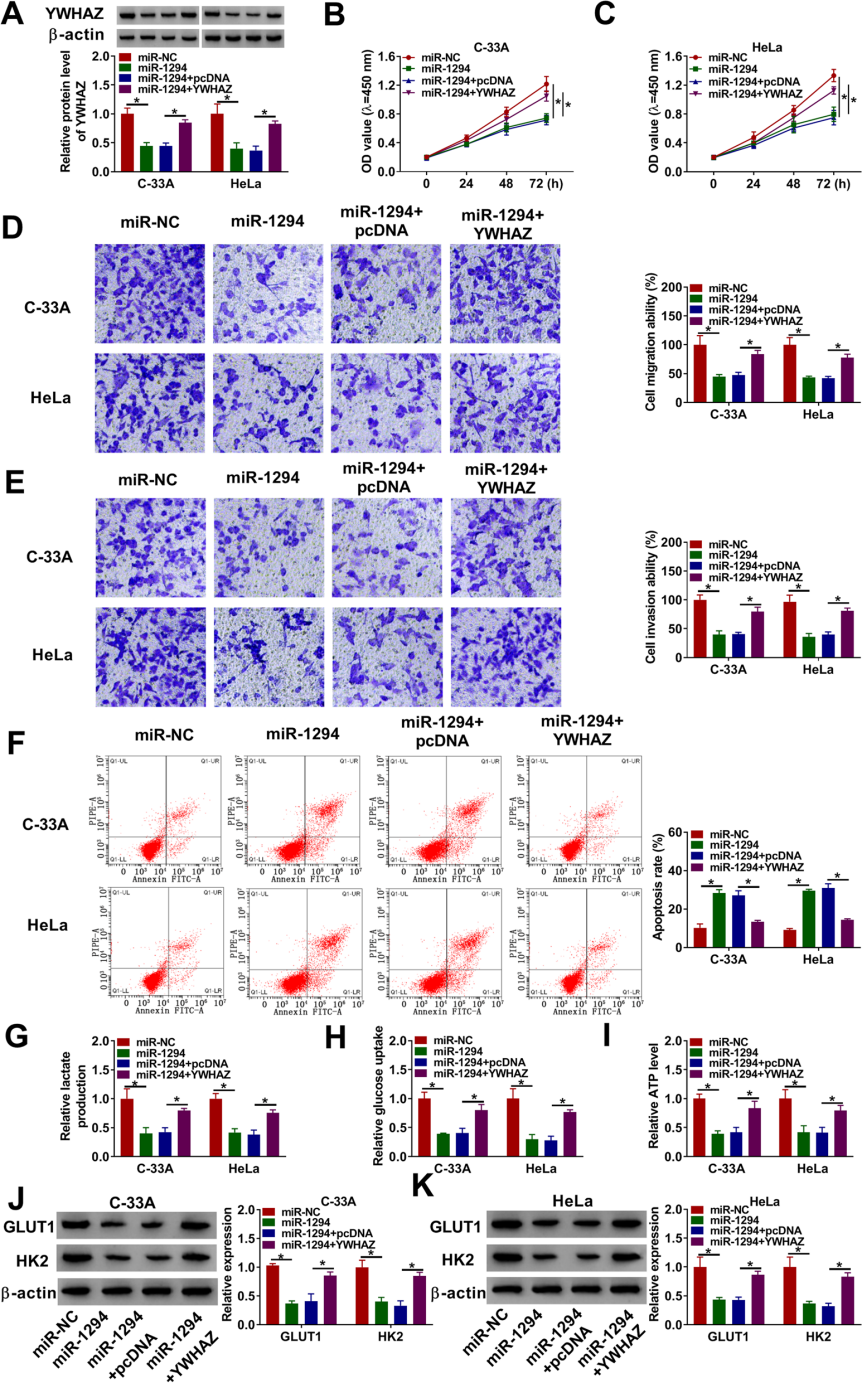
MiR-1294 mimic inhibited CC process by binding to YWHAZ.** A** Western blot was employed to illustrate the effects between miR-1294 mimic and YWHAZ on YWHAZ protein expression in C-33A and HeLa cells. **B** and **C** The impacts between miR-1294 and YWHAZ on the proliferation of C-33A and HeLa cells were investigated by CCK-8 assay. **D** and **E** The influences between miR-1294 mimic and enforced YWHAZ expression on the migration and invasion of C-33A and HeLa cells were demonstrated by transwell assay. **F** Flow cytometry analysis was performed to elucidate the impacts between miR-1294 and YWHAZ on cell apoptosis in C-33A and HeLa cells. **G** Lactate assay kit was conducted to demonstrate the effects between miR-1294 and YWHAZ on lactate production in C-33A and HeLa cells. **H** Glucose assay kit was used to determine the impacts between miR-1294 mimic and YWHAZ on glucose uptake in C-33A and HeLa cells. **I** The effects between miR-1294 and ectopic YWHAZ expression on ATP level were presented by ATP detection kit in C-33A and HeLa cells. **J** and **K** Western blot was carried out to elaborate the effects between miR-1294 and circCDK17 on the protein expression levels of GLUT1 and HK2 in C-33A and HeLa cells. *P < 0.05

Given that miR-1294 was associated with YWHAZ, whether miR-1294 regulated CC process via associating with YWHAZ was further disclosed. The effects between miR-1294 mimic and YWHAZ overexpression on YWHAZ expression were firstly unveiled. Data showed that YWHAZ overexpression restored the inhibition effect of miR-1294 on YWHAZ protein expression (Fig. 6A). Subsequently, CCK-8 assay elucidated that miR-1294 mimic inhibited the proliferation of C-33A and HeLa cells, whereas YWHAZ overexpression abolished this effect (Fig. 6B and C). The migratory and invasive abilities of C-33A and HeLa cells were also repressed after miR-1294 mimic transfection, which was hindered by YWHAZ overexpression (Fig. 6D and E). MiR-1294 mimic promoted the apoptosis of C-33A and HeLa cells; however, ectopic YWHAZ expression restrained this effect (Fig. 6F). Additionally, miR-1294 mimic repressed lactate production, while YWHAZ overexpression reversed this result (Fig. 6G). MiR-1294 mimic inhibited glucose uptake and decreased ATP level in C-33A and HeLa cells; but these results were impaired after YWHAZ overexpression (Fig. 6H and I). Furthermore, the protein expression levels of GLUT1 and HK2 were down-regulated by miR-1294 mimic in C-33A and HeLa cells, whereas YWHAZ overexpression blocked these influences (Fig. 6J and K). These results uncovered that miR-1294 repressed tumor development and glycolysis via associating with YWHAZ in CC.

### CircCDK17 knockdown repressed tumor formation in vivo

**Fig. 7:**
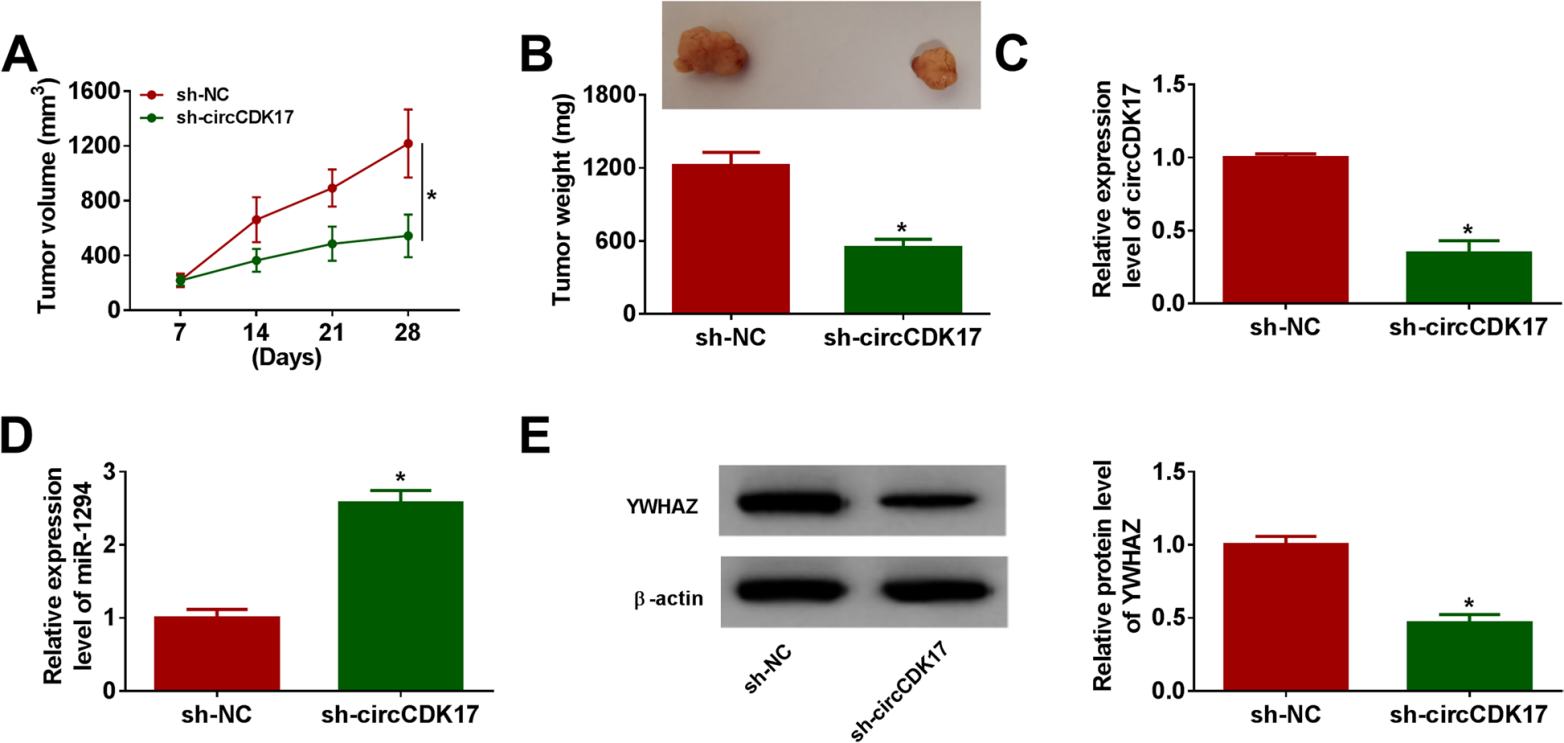
CircCDK17 silencing suppressed tumor growth in vivo.** A** and **B** The effects of circCDK17 knockdown on the volume and weight of tumors were illustrated. **C** and **D** QRT-PCR was performed to illustrate the impacts of circCDK17 silencing on the expression levels of circCDK17 and miR-1294 in vivo. **E** The effect of circCDK17 silencing on YWHAZ protein expression in vivo was disclosed by western blot. *P < 0.05

The effect of circCDK17 on tumor growth was further demonstrated in vivo. Results showed that circCDK17 silencing decreased tumor volume and reduced tumor weight (Fig. 7A and B). Additionally, whether circCDK17 affected the expression levels of miR-1294 and YWHAZ in vivo was continued to reveal. Result firstly illustrated circCDK17 was successfully knocked down (Fig. 7C). Subsequently, circCDK17 silencing dramatically increased miR-1294 expression and decreased YWHAZ protein expression in vivo (Fig. 7D and E). The above data implicated circCDK17 silencing suppressed tumor formation by regulating miR-1294 and YWHAZ in vivo.

## Discussion

CC is a tumor in reproductive system and accounts for more than half causes of cancer-related deaths [23]. Despite the incidence and fatality rate of CC are obviously decreased owing to much process in technology for CC screening, the prognosis of advanced CC cases also remains dissatisfactory [24]. CircRNA is found that it can regulate CC progression. As presented in current study, circ_0000745 enhanced cell abilities in proliferation, migration and invasion in CC [10]; circ_0001038, a highly expressed circRNA in CC tissues and cells, was closely correlated with the lymphatic metastasis of CC patients [25]. Circ_0060467 participated in the process of epithelial-mesenchymal transition (EMT) [26] and circ_0100129 (circ-ATP8A2) silencing repressed cell proliferation and metastasis, while facilitated cell apoptosis in CC [27]. However, the effects and mechanism of circCDK17 in modulating CC process are unknown.

In this study, circCDK17 was found to be enrolled in CC development for the first time. In the current study, in order to find the differently expressed circRNAs in CC tissues, GSE102686 database was performed. Results showed that the change of circ_101119 (circCDK17) expression was the most obvious among differently expressed circRNAs. And circCDK17 was chosen for further study. Subsequently, results showed that circCDK17 knockdown attenuated cell proliferation, migration and invasion, whereas facilitated cell apoptosis in CC. Glycolysis is called as Warburg effect and is involved in the conversion of glucose to lactate under aerobic microenvironment [28]. And glycolysis is very helpful to cell proliferation in cancers owing to the production of lots of lipids, proteins, and nucleotides [29]. Therefore, the impact of circCDK17 silencing on glycolysis was continued to explore. Results showed that circCDK17 knockdown hindered glycolysis. Our results also displayed that circCDK17 mainly existed in nucleus, implicating circRNA participated in the regulation at post-transcriptional level. Furthermore, xenograft mouse model assay presented that circCDK17 downregulation decreased tumor volume and reduced tumor weight.

Emerging evidences illustrated circRNA could regulate cancer progression via sponging miRNA [30]. Thus, the miRNA bound to circCDK17 was predicted. Results showed that circCDK17 was associated with miR-1294. Previous research explained miR-1294 was lowly expressed and was related to poor prognosis in epithelial ovarian cancer [31]. Besides, Zhang et al. demonstrated that circ_0004370 knockdown hindered cell proliferation and invasion, whereas contributed to cell apoptosis through binding to miR-1294 in esophageal cancer [32]. Other like, miR-1294 mimic suppressed cell proliferation and invasion in osteosarcoma [17]. The above data meant that miR-1294 acted as a tumor suppressor. In this study, we found miR-1294 expression was dramatically downregulated in CC tissues and cells. And miR-1294 inhibitor attenuated the inhibition effects of circCDK17 silencing on cell proliferation, migration, invasion and glucose metabolism, and the promotion impact of that on cell apoptosis in CC. These findings suggested miR-1294 also functioned as a repressor in CC process and circCDK17 regulated CC progression through associating with miR-1294.

Growing evidences showed that YWHAZ expression was commonly increased in lots of cancers and YWHAZ mediated metastasis-related protein expression [33]. In addition, YWHAZ might improve apoptosis resistance via regulating apoptosis-related pathways [34]. These evidences suggested YWHAZ played a key part in tumor progression. Ji et al. have investigated that SNHG14 silencing suppressed cell proliferation and migratory and invasive abilities, whereas promoted cell apoptosis; however, these effects were partly abolished by enforced YWHAZ expression in CC [22], suggesting that YWHAZ could enhance cell proliferative, migratory and invasive abilities, while repress cell apoptosis in CC. Coincidently, YWHAZ was found that it could bind to miR-1294 in our study. Additionally, recuse experiments presented YWHAZ overexpression restored the effects of miR-1294 mimic on cell proliferation, migration, invasion and apoptosis, meaning that YWHAZ contributed to CC progression. This finding was consistent with the above data. Besides, YWHAZ was showed that it could promote glucose metabolism.

## Conclusion

All in all, circCDK17 and YWHAZ expression levels were obviously upregulated, while miR-1294 expression was apparently downregulated in CC tissues or cells. Additionally, circCDK17 silencing repressed cell proliferation, migration, invasion and glucose metabolism, whereas induced cell apoptosis via downregulating YWHAZ expression through binding to miR-1294 in CC. Furthermore, circCDK17 knockdown hindered tumor formation of CC in vivo. These findings provide a new insight to study circRNA-mediated therapy in CC.

## Data Availability

Data analyzed for this study will be available on a reasonable request
